# Supplementary material for: Investigation of Weather Triggers Preceding Outbreaks of Acute Bovine Liver Disease in Australia
Source: Toxins (Basel). 2022 Jun 17;14(6):414. doi: 10.3390/toxins14060414 (PMC9229311; doi:10.3390/toxins14060414)
Supplement: Supplementary file 1 [file toxins-14-00414-s001.zip › toxins-1750373 - Supplementary - Final.pdf]

toxins

MDPI

# Investigation of Weather Triggers Preceding Outbreaks of Acute Bovine Liver Disease in Australia

**Eve M. Manthorpe, Grant T. Rawlin, Mark A. Stevenson, Lucy Woolford and Charles G. B. Caraguel**

**Table S1.** Weather dataset for the 15-days preceding an outbreak of acute bovine liver disease in the outbreak year (0) and two preceding years (-1, -2) when no outbreak occurred, inclusive of property and outbreak identification, and outbreak date. Abbreviations: avg, average; cum, cumulative; DP, dewpoint (degrees Celsius; °C); OID, outbreak identification; Odate, outbreak date; PID, property identification; prec, precipitation (millimetres; mm); sol, solar exposure (Joule(s) per square metre; MJ/m$^2$); Tmax, maximum temperature (degrees Celsius; °C); Tmin, minimum temperature (degrees Celsius; °C); Tmin-DP, average of daily minimum and dewpoint at 9am temperature gap (degrees Celsius; °C).

| PID | OID | Odate | Case | Year | Tmin (avg) | Tmax (avg) | Prec (avg) | Prec (cum) | Sol (avg) | Sol (cum) | DP 0900 (avg) | DP 1500 (avg) | Tmin-DP | Tmax-DP |
|---|---|---|---|---|---|---|---|---|---|---|---|---|---|---|
| A | 1 | 20/2/2008 | 1 | 0 | 8.55 | 20.46 | 0.49 | 7.42 | 12.03 | 180.49 | 10.69 | 9.71 | -2.14 | 10.75 |
| A | 1 | 20/2/2008 | 0 | -1 | 9.63 | 22.82 | 0.08 | 1.25 | 12.97 | 194.57 | 9.77 | 8.82 | -0.14 | 14.00 |
| A | 1 | 20/2/2008 | 0 | -2 | 8.71 | 17.29 | 1.96 | 29.41 | 9.56 | 133.90 | 8.17 | 7.57 | 0.54 | 9.72 |
| B | 2 | 17/5/2017 | 1 | 0 | 9.49 | 18.02 | 0.73 | 10.96 | 8.23 | 123.42 | 10.10 | 10.26 | -0.61 | 7.76 |
| B | 2 | 17/5/2017 | 0 | -1 | 11.45 | 19.13 | 1.45 | 21.70 | 7.18 | 107.67 | 11.70 | 12.10 | -0.25 | 7.02 |
| B | 2 | 17/5/2017 | 0 | -2 | 8.86 | 15.65 | 3.27 | 49.03 | 6.60 | 92.39 | 9.24 | 9.58 | -0.38 | 6.07 |
| C | 3 | 31/3/2010 | 1 | 0 | 13.06 | 23.71 | 1.09 | 16.35 | 12.66 | 189.93 | 12.83 | 13.09 | 0.23 | 10.62 |
| C | 3 | 31/3/2010 | 0 | -1 | 10.21 | 23.21 | 0.54 | 8.09 | 15.80 | 236.98 | 10.34 | 9.65 | -0.13 | 13.56 |
| C | 3 | 31/3/2010 | 0 | -2 | 11.34 | 23.95 | 1.63 | 24.51 | 14.42 | 216.32 | 10.08 | 9.45 | 1.26 | 14.51 |
| D | 4 | 1/4/2010 | 1 | 0 | 13.43 | 24.33 | 1.66 | 24.97 | 12.60 | 189.06 | 13.48 | 13.42 | -0.06 | 10.91 |
| D | 4 | 1/4/2010 | 0 | -1 | 11.87 | 23.42 | 4.23 | 63.40 | 17.05 | 255.82 | 13.24 | 12.71 | -1.38 | 10.71 |
| D | 4 | 1/4/2010 | 0 | -2 | 12.72 | 23.18 | 1.66 | 24.97 | 13.61 | 204.14 | 12.67 | 13.04 | 0.04 | 10.14 |
| E | 5 | 20/4/2010 | 1 | 0 | 12.37 | 19.82 | 2.25 | 33.79 | 8.60 | 128.95 | 12.08 | 12.49 | 0.29 | 7.33 |
| E | 5 | 20/4/2010 | 0 | -1 | 8.92 | 18.90 | 1.26 | 18.85 | 12.01 | 180.22 | 10.55 | 10.10 | -1.62 | 8.80 |
| E | 5 | 20/4/2010 | 0 | -2 | 9.22 | 20.56 | 0.59 | 8.91 | 12.43 | 186.44 | 11.11 | 10.38 | -1.90 | 10.19 |
| E | 6 | 20/4/2010 | 1 | 0 | 13.05 | 20.27 | 2.24 | 33.58 | 8.56 | 128.40 | 12.33 | 12.81 | 0.73 | 7.46 |
| E | 6 | 20/4/2010 | 0 | -1 | 9.73 | 19.27 | 1.31 | 19.67 | 12.19 | 182.87 | 10.84 | 10.47 | -1.12 | 8.80 |
| E | 6 | 20/4/2010 | 0 | -2 | 9.99 | 20.96 | 0.64 | 9.61 | 12.62 | 189.29 | 11.40 | 10.79 | -1.41 | 10.17 |
| F | 7 | 23/4/2010 | 1 | 0 | 12.03 | 20.39 | 1.65 | 24.74 | 8.91 | 133.67 | 12.37 | 12.61 | -0.34 | 7.79 |
| F | 7 | 23/4/2010 | 0 | -1 | 8.14 | 20.62 | 0.82 | 12.31 | 13.10 | 196.52 | 10.19 | 9.51 | -2.06 | 11.12 |
| F | 7 | 23/4/2010 | 0 | -2 | 8.44 | 20.95 | 0.22 | 3.24 | 11.75 | 176.31 | 10.61 | 9.67 | -2.17 | 11.28 |
| G | 8 | 10/3/2011 | 1 | 0 | 12.98 | 21.44 | 1.15 | 17.28 | 17.01 | 255.15 | 11.45 | 12.92 | 1.53 | 8.52 |
| G | 8 | 10/3/2011 | 0 | -1 | 13.30 | 22.40 | 2.16 | 32.38 | 17.79 | 266.89 | 12.25 | 12.80 | 1.05 | 9.61 |
| G | 8 | 10/3/2011 | 0 | -2 | 12.03 | 22.66 | 0.85 | 12.78 | 17.23 | 258.45 | 10.66 | 11.28 | 1.37 | 11.38 |

| | | | | | | | | | | | | | | |
|---|---|---|---|---|---|---|---|---|---|---|---|---|---|---|
| H | 9 | 26/4/2011 | 1 | 0 | 9.73 | 18.08 | 5.61 | 84.14 | 10.25 | 153.69 | 11.75 | 12.08 | -2.01 | 6.00 |
| H | 9 | 26/4/2011 | 0 | -1 | 13.01 | 20.37 | 2.13 | 31.91 | 9.27 | 139.00 | 12.14 | 12.59 | 0.87 | 7.78 |
| H | 9 | 26/4/2011 | 0 | -2 | 9.74 | 20.62 | 2.15 | 32.21 | 11.53 | 173.02 | 10.42 | 10.42 | -0.68 | 10.20 |
| I | 10 | 21/5/2013 | 1 | 0 | 8.86 | 19.62 | 1.55 | 23.30 | 9.94 | 149.17 | 8.79 | 7.46 | 0.07 | 12.16 |
| I | 10 | 21/5/2013 | 0 | -1 | 8.59 | 17.67 | 1.88 | 28.26 | 8.33 | 124.99 | 8.83 | 8.67 | -0.24 | 9.00 |
| I | 10 | 21/5/2013 | 0 | -2 | 8.22 | 15.30 | 2.67 | 40.01 | 6.41 | 96.09 | 7.62 | 7.83 | 0.60 | 7.47 |
| J | 11 | 9/6/2013 | 1 | 0 | 8.19 | 15.72 | 2.17 | 32.61 | 5.88 | 88.14 | 9.40 | 9.72 | -1.21 | 6.00 |
| J | 11 | 9/6/2013 | 0 | -1 | 7.03 | 13.77 | 3.11 | 46.63 | 5.25 | 78.79 | 8.11 | 8.61 | -1.08 | 5.15 |
| J | 11 | 9/6/2013 | 0 | -2 | 5.66 | 13.88 | 1.69 | 25.30 | 5.97 | 89.50 | 7.07 | 7.54 | -1.41 | 6.34 |
| J | 12 | 6/7/2013 | 1 | 0 | 5.46 | 14.49 | 0.97 | 14.49 | 6.18 | 92.74 | 6.88 | 8.16 | -1.42 | 6.33 |
| J | 12 | 6/7/2013 | 0 | -1 | 5.40 | 12.05 | 3.09 | 46.29 | 5.57 | 83.59 | 6.52 | 7.15 | -1.12 | 4.90 |
| J | 12 | 6/7/2013 | 0 | -2 | 6.17 | 14.20 | 3.22 | 48.37 | 5.86 | 87.84 | 6.72 | 8.06 | -0.54 | 6.14 |
| K | 13 | 19/4/2014 | 1 | 0 | 8.08 | 16.70 | 9.57 | 143.60 | 9.04 | 135.65 | 10.01 | 10.56 | -1.93 | 6.15 |
| K | 13 | 19/4/2014 | 0 | -1 | 7.72 | 19.07 | 1.13 | 16.99 | 11.98 | 179.69 | 9.22 | 9.85 | -1.48 | 9.23 |
| K | 13 | 19/4/2014 | 0 | -2 | 6.85 | 18.66 | 0.72 | 10.74 | 14.60 | 219.04 | 8.56 | 9.24 | -1.36 | 9.42 |
| L | 14 | 24/4/2014 | 1 | 0 | 10.49 | 19.56 | 2.57 | 38.50 | 9.55 | 143.25 | 12.19 | 11.28 | -1.71 | 8.27 |
| L | 14 | 24/4/2014 | 0 | -1 | 8.59 | 20.43 | 0.57 | 8.50 | 10.97 | 164.48 | 10.21 | 8.24 | -1.61 | 12.19 |
| L | 14 | 24/4/2014 | 0 | -2 | 10.08 | 21.88 | 0.89 | 13.31 | 12.42 | 186.29 | 10.92 | 9.32 | -0.80 | 12.71 |
| M | 15 | 25/4/2014 | 1 | 0 | 11.13 | 17.96 | 2.62 | 39.28 | 10.46 | 156.90 | 11.74 | 11.08 | -0.61 | 6.89 |
| M | 15 | 25/4/2014 | 0 | -1 | 9.80 | 18.41 | 1.03 | 15.48 | 9.77 | 146.61 | 9.94 | 8.99 | -0.14 | 9.42 |
| M | 15 | 25/4/2014 | 0 | -2 | 11.11 | 20.01 | 2.44 | 36.60 | 11.30 | 169.52 | 11.36 | 10.41 | -0.20 | 9.61 |
| N | 16 | 4/6/2014 | 1 | 0 | 8.98 | 17.34 | 2.04 | 30.54 | 5.82 | 87.33 | 11.28 | 11.87 | -2.30 | 5.48 |
| N | 16 | 4/6/2014 | 0 | -1 | 7.19 | 15.90 | 1.85 | 27.78 | 5.91 | 88.71 | 9.08 | 9.61 | -1.89 | 6.29 |
| N | 16 | 4/6/2014 | 0 | -2 | 6.76 | 14.70 | 2.57 | 38.60 | 5.91 | 88.61 | 7.99 | 8.19 | -1.23 | 6.50 |
| O | 17 | 20/4/2015 | 1 | 0 | 9.92 | 19.23 | 1.81 | 27.16 | 11.46 | 171.90 | 11.65 | 11.02 | -1.73 | 8.21 |
| O | 17 | 20/4/2015 | 0 | -1 | 12.53 | 20.26 | 2.18 | 32.68 | 10.75 | 161.22 | 13.41 | 13.15 | -0.87 | 7.11 |
| O | 17 | 20/4/2015 | 0 | -2 | 10.61 | 20.39 | 0.78 | 11.70 | 12.12 | 181.80 | 11.93 | 11.85 | -1.52 | 8.54 |
| P | 18 | 23/11/2017 | 1 | 0 | 10.62 | 24.75 | 0.35 | 5.26 | 24.95 | 374.18 | 13.00 | 12.43 | -2.39 | 12.33 |
| P | 18 | 23/11/2017 | 0 | -1 | 9.90 | 20.73 | 1.64 | 24.56 | 19.67 | 295.04 | 10.68 | 10.89 | -0.78 | 9.84 |
| P | 18 | 23/11/2017 | 0 | -2 | 9.20 | 22.15 | 1.59 | 23.82 | 20.60 | 309.01 | 10.93 | 10.99 | -1.72 | 11.16 |
| Q | 19 | 21/4/2018 | 1 | 0 | 10.43 | 22.47 | 1.78 | 26.67 | 11.07 | 166.06 | 10.94 | 10.12 | -0.52 | 12.35 |
| Q | 19 | 21/4/2018 | 0 | -1 | 9.58 | 20.49 | 1.81 | 27.20 | 10.16 | 152.35 | 9.81 | 10.14 | -0.23 | 10.36 |
| Q | 19 | 21/4/2018 | 0 | -2 | 10.00 | 20.27 | 1.45 | 21.72 | 10.80 | 161.98 | 11.15 | 10.60 | -1.16 | 9.67 |
| R | 20 | 21/5/2018 | 1 | 0 | 7.61 | 14.97 | 4.33 | 64.96 | 6.81 | 102.14 | 9.11 | 8.42 | -1.50 | 6.55 |
| R | 20 | 21/5/2018 | 0 | -1 | 5.60 | 15.58 | 1.20 | 18.02 | 7.42 | 111.30 | 8.73 | 9.34 | -3.13 | 6.24 |
| R | 20 | 21/5/2018 | 0 | -2 | 10.19 | 18.01 | 4.47 | 67.05 | 7.40 | 111.02 | 11.06 | 10.84 | -0.87 | 7.17 |
| S | 21 | 21/5/2018 | 1 | 0 | 7.61 | 14.97 | 4.33 | 64.96 | 6.81 | 102.14 | 9.11 | 8.42 | -1.50 | 6.55 |
| S | 21 | 21/5/2018 | 0 | -1 | 5.60 | 15.58 | 1.20 | 18.02 | 7.42 | 111.30 | 8.73 | 9.34 | -3.13 | 6.24 |
| S | 21 | 21/5/2018 | 0 | -2 | 10.19 | 18.01 | 4.47 | 67.05 | 7.40 | 111.02 | 11.06 | 10.84 | -0.87 | 7.17 |
| T | 22 | 26/9/2018 | 1 | 0 | 4.55 | 16.31 | 1.06 | 15.83 | 15.20 | 227.97 | 5.45 | 4.92 | -0.90 | 11.38 |
| T | 22 | 26/9/2018 | 0 | -1 | 5.91 | 17.15 | 3.08 | 46.21 | 15.42 | 231.27 | 7.04 | 6.74 | -1.13 | 10.42 |
| T | 22 | 26/9/2018 | 0 | -2 | 7.00 | 15.28 | 1.40 | 20.94 | 12.70 | 190.48 | 8.20 | 7.82 | -1.20 | 7.46 |

| | | | | | | | | | | | | | | |
|---|---|---|---|---|---|---|---|---|---|---|---|---|---|---|
| U | 23 | 15/4/2019 | 1 | 0 | 9.38 | 21.64 | 0.85 | 12.71 | 12.52 | 187.76 | 10.20 | 10.10 | -0.83 | 11.54 |
| U | 23 | 15/4/2019 | 0 | -1 | 11.56 | 25.54 | 0.11 | 1.67 | 13.45 | 201.70 | 11.43 | 10.67 | 0.13 | 14.87 |
| U | 23 | 15/4/2019 | 0 | -2 | 8.63 | 21.40 | 4.95 | 74.22 | 12.10 | 181.48 | 10.01 | 9.33 | -1.38 | 12.07 |
| V | 24 | 4/5/2019 | 1 | 0 | 9.04 | 21.72 | 6.57 | 98.50 | 11.31 | 169.59 | 10.57 | 10.27 | -1.54 | 11.44 |
| V | 24 | 4/5/2019 | 0 | -1 | 7.66 | 23.05 | 0.20 | 3.03 | 12.59 | 188.90 | 8.51 | 8.06 | -0.85 | 14.99 |
| V | 24 | 4/5/2019 | 0 | -2 | 7.49 | 18.67 | 3.91 | 58.58 | 9.99 | 149.88 | 9.59 | 9.55 | -2.10 | 9.12 |
| U | 25 | 5/7/2019 | 1 | 0 | 3.38 | 15.46 | 0.28 | 4.25 | 8.38 | 125.69 | 4.22 | 5.31 | -0.84 | 10.16 |
| U | 25 | 5/7/2019 | 0 | -1 | 2.69 | 14.60 | 0.17 | 2.61 | 7.80 | 117.04 | 3.12 | 5.22 | -0.43 | 9.37 |
| U | 25 | 5/7/2019 | 0 | -2 | 3.06 | 14.53 | 0.33 | 4.98 | 7.54 | 113.06 | 3.87 | 4.76 | -0.80 | 9.76 |
| W | 26 | 20/4/2020 | 1 | 0 | 9.34 | 18.17 | 2.10 | 31.51 | 10.24 | 153.65 | 10.37 | 9.29 | -1.04 | 8.88 |
| W | 26 | 20/4/2020 | 0 | -1 | 10.06 | 22.30 | 0.58 | 8.73 | 11.56 | 173.46 | 9.67 | 8.04 | 0.38 | 14.26 |
| W | 26 | 20/4/2020 | 0 | -2 | 10.39 | 22.49 | 1.67 | 25.01 | 10.59 | 158.84 | 10.98 | 9.20 | -0.59 | 13.29 |

**Table S2.** Correlation matrix across the weather parameters summarised over a 15-day prior the report of an acute bovine liver disease event or the same calendar date the one or two years prior. Reported estimates are pairwise Pearson's correlation coefficient (*r*, ranging between -1 and +1).
Abbreviations: avg, average; cum, cumulative; DP, dewpoint (degrees Celsius; °C); OID, outbreak identification; Odate, outbreak date; PID, property identification; prec, precipitation (millimetres; mm); RH, relative humidity (per cent; %); sol. exp, solar exposure (Joule(s) per square metre; $MJ/m^2$); Tavg, average temperature (degrees Celsius; °C); Tgap, average temperature gap (Tmax-Tmin); Tmax, maximum temperature (degrees Celsius; °C); Tmin, minimum temperature (degrees Celsius; °C); Tmin-DP, average of daily minimum and dewpoint at 9am temperature gap (degrees Celsius; °C); vap. press, vapour pressure (hectopascals; hPa).

| | $Avg.daily\ T_{min}$ | $Avg.daily\ T_{max}$ | $Avg.daily\ T_{avg}$ | $Avg.daily\ T_{gap}$ | $Avg.daily\ vap.press_{3pm}$ | $Cum.daily\ prec.$ | $Avg.daily\ sol.exp$ | $Cum.daily\ sol.exp$ | $Avg.daily\ RH_{9am}$ | $Avg.daily\ RH_{3pm}$ | $Avg.DP_{9am}$ | $Avg.DP_{3pm}$ | $Avg.min.to\ 9am\ DP_{gap}$ | $Avg.max.to\ 3pm\ DP_{gap}$ |
|---|---|---|---|---|---|---|---|---|---|---|---|---|---|---|
| $Avg.daily\ T_{min}$ | - | | | | | | | | | | | | | |
| $Avg.daily\ T_{max}$ | 0.7380* | - | | | | | | | | | | | | |
| $Avg.daily\ T_{avg}$ | 0.9144* | 0.9480* | - | | | | | | | | | | | |
| $Avg.daily\ T_{gap}$ | -0.0723 | 0.6197* | 0.3376* | - | | | | | | | | | | |
| $Avg.daily\ vap.press_{3pm}$ | 0.8756* | 0.5836* | 0.7632* | -0.1558 | - | | | | | | | | | |
| $Cum.daily\ prec.$ | 0.0295 | -0.2479* | -0.1346 | -0.4004* | 0.1319 | - | | | | | | | | |
| $Avg.daily\ sol.exp$ | 0.3837* | 0.7174* | 0.6114* | 0.6143* | 0.3275* | -0.2576* | - | | | | | | | |
| $Cum.daily\ sol.exp$ | 0.3832* | 0.7185* | 0.6118* | 0.6165* | 0.3293* | -0.2582* | 0.9997* | - | | | | | | |
| $Avg.daily\ RH_{9am}$ | -0.4764* | -0.2708* | -0.3873* | 0.154 | -0.1817 | 0.1098 | -0.0869 | -0.0827 | - | | | | | |
| $Avg.daily\ RH_{3pm}$ | -0.5994* | -0.5304* | -0.6008* | -0.0868 | -0.1574 | 0.1427 | -0.2540* | -0.2500* | 0.7299* | - | | | | |
| $Avg.DP_{9am}$ | 0.9165* | 0.7024* | 0.8537* | -0.0276 | 0.9201* | 0.1022 | 0.3832* | 0.3845* | -0.0901 | -0.3310* | - | | | |
| $Avg.DP_{3pm}$ | 0.8725* | 0.5810* | 0.7602* | -0.1561 | 0.9958* | 0.1393 | 0.3135* | 0.3154* | -0.1481 | -0.137 | 0.9326* | - | | |
| $Avg.min.to\ 9am\ DP_{gap}$ | 0.4882* | 0.3055* | 0.4136* | -0.1166 | 0.1728 | -0.1509 | 0.124 | 0.1199 | -0.9892* | -0.7521* | 0.0988 | 0.1375 | - | |
| $Avg.max.to\ 3pm\ DP_{gap}$ | 0.2164 | 0.7670* | 0.5621* | 0.8819* | -0.07 | -0.4135* | 0.6316* | 0.6316* | -0.2144 | -0.5417* | 0.1257 | -0.0765 | 0.2655* | - |

Correlation estimates with a star (*) were significantly different from *r* = 0.00 at the 5% significance level. Strong ($r \geq 0.67$) significant ($P < 0.05$) positive ($r > 0.00$) correlations are reported in green, mild to moderate ($0 > r < 0.67$) significant ($P < 0.05$) positive ($r > 0.00$) correlations in clear green, strong ($r \leq -0.67$) significant ($P < 0.05$) negative ($r < 0.00$) correlations are reported in dark red, mild to moderate ($-0.67 > r < 0$) significant ($P < 0.05$) negative ($r < 0.00$) correlations in weak orange, and non-significant correlation ($P > 0.05$) correlation in gray.
